# Hospital Festivals, Meetings, &c.

**Published:** 1895-10-05

**Authors:** 


### HOSPITAL FESTIVALS, MEETINGS, &C.

### CAXTON CONVALESCENT HOME, LIMPSFIELD.

On Saturday last the central block of the new convalescent home which has, chiefly through the liberality of Mr. Passmore Edwards, been erected upon an ideal site on the beautiful Limpsfield Chart, for the benefit of the "Printing and Allied Trades," was formally opened by Mr. Passmore Edwards himself. The ground originally formed part of the site secured for another charitable undertaking by Mr. Edwards, a convalescent home in connection with Charing Cross Hospital, now in course of building not many hundred yards away. Nine acres of this land have been purchased by the trustees from the hospital authorities and put aside permanently for the Caxton Home.

The home when completed will contain about 70 beds, but as yet only a portion has been finished to accommodate about 20 patients, at a cost of £3,600. A further £5,000 will be needed to complete the two wings included in the plans. The architect is Mr. A. S. Snell.

A good many friends of the institution journeyed down to Limpsfield on Saturday for the opening ceremony, before which Mrs. Passmore Edwards unveiled a medallion portrait of her husband, as president of the institution, placed over the front entrance. Mr. C. J. Drummond, vice-president, in a short speech, stated that the building once erected, the cost of maintenance would be easily forthcoming. He considered printers were probably the most provident section of the working classes of the country; there were few in the trade who did not subscribe on an average half-a-crown per week out of their wages for provident purposes. Mr. Edwards, in reply to a vote of thanks, referred to the fact that the home was the absolute property of the members of the trade without regard to any question of belonging to trade organisations, or of religious or political faith.

Amongst other gifts and benefactions Mr. Passmore Edwards has given 500 volumes towards the formation of a library, a wise charity, which it may be hoped will be augmented by other friends in due course.

